# 2609. Comprehensive cost assessment among families with children younger than 2 years-of-age hospitalized with RSV infection

**DOI:** 10.1093/ofid/ofad500.2223

**Published:** 2023-11-27

**Authors:** Krow Ampofo, Evan G Heller, Lazarus Adua, Abbey Lovebridge, Kaleb Miller, Kathrine Lindsey Werdan, Per H Gesteland, Madelyn Ruggieri, Lyn Finelli, Yoonyoung Choi

**Affiliations:** University of Utah, Salt Lake City, Utah; University of Utah, Salt Lake City, Utah; University of Utah, Salt Lake City, Utah; University of Utah, Salt Lake City, Utah; University of Utah, Salt Lake City, Utah; University of Utah, Salt Lake City, Utah; University of Utah School of Medicine, Salt Lake City, Utah; Merck & Co., Inc., Rahway, NJ, Philadelphia, Pennsylvania; Merck&Co., Rose Valley, Pennsylvania; Merck, Philadelphia, Pennsylvania

## Abstract

**Background:**

Respiratory Syncytial Virus (RSV) is a major cause of childhood lower respiratory tract infection (LRTI) in the U.S. While RSV hospital (direct) cost is well described, the impact on work productivity and additional RSV-related (indirect) costs are not known. We describe direct and indirect cost occurring to a family before, during, and after child’s RSV hospitalization.

**Figure:**
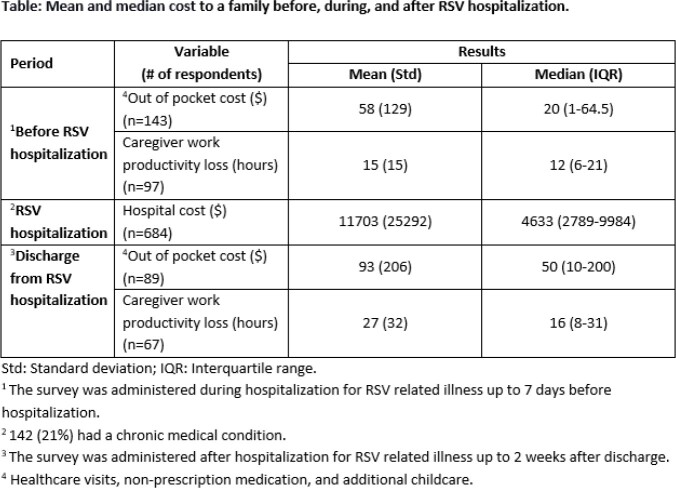

**Methods:**

We prospectively identified children < 2 years of age hospitalized with laboratory-confirmed RSV LRTI at Primary Children’s and Riverton Hospitals, Salt Lake City, UT, from 12/2019 to 5/2022. RSV hospitalization-related cost consisted of 1) Hospital cost, 2) indirect costs occurring between a week before admission and up to 2 weeks post discharge, and 3) productivity loss of caregivers during child’s admission. We abstracted hospital financial data and surveyed caregivers using a questionnaire developed for the study, along with the Work Productivity and Activity Impairment Questionnaire: Child’s Hospitalization for Respiratory Illness instrument.

**Results:**

During the study period, 684 children < 2 years were hospitalized with RSV LRTI with a median length of stay 1.8 days (IQR 1-3.1 days) and mean hospital cost of 2023 USD $11703 (std: $25292). Of the 146 (21%) caregivers who participated in the survey, 140/146 (96%) reported at least one caregiver is currently employed and 123/146 (79%) were female. One week prior to admission, 109/146 (75%) visited a healthcare facility and spent a mean of $58 (std $129) out of pocket cost for the visits, medication, and additional childcare. Before RSV hospitalization, 81/140 (58%) employed caregivers lost work hours (hrs) (mean 15 hrs; std 15 hrs) and had an average productivity impairment score of 4.4 out of 10 (std 3.6) (10=worst impairment). After discharge, 68/146 (47%) children required a healthcare visit, 67/140 (48%) caregivers lost work hrs (mean 27 hrs; std 32 hrs) and spent a mean of $93 (std $206) out of pocket cost. (Table)

**Conclusion:**

The hospitalization of children < 2 years with RSV LRTI is associated with significant costs during and after hospitalization. These costs include caregivers’ loss of work hours and additional out-of-pocket costs. Our data support the need for RSV vaccines and immunoprophylaxis to prevent RSV hospitalization.

**Disclosures:**

**Krow Ampofo, MBChB**, Merck: Advisor/Consultant|Merck: Grant/Research Support **Lyn Finelli, DrPH, MS**, Merck&Co: Stocks/Bonds **Yoonyoung Choi, PhD, MS, RPh**, Merck: Employed|Merck: Stocks/Bonds

